# Benchmarking electrical methods for rapid estimation of root biomass

**DOI:** 10.1186/s13007-016-0133-7

**Published:** 2016-06-22

**Authors:** François Postic, Claude Doussan

**Affiliations:** ARVALIS Institut du végétal, 75116 Paris, France; UMR EMMAH, INRA, UAPV, Domaine Saint-Paul, Site Agroparc, 84914 Avignon, France

**Keywords:** Wheat, *Triticum durum*, Root mass, Electrical impedance spectrometry, Capacitance, Terminal configuration, Contact electrode

## Abstract

**Background:**

To face climate change and subsequent rainfall instabilities, crop breeding strategies now include root traits phenotyping. Rapid estimation of root traits in controlled conditions can be achieved by using parallel electrical capacitance and its linear correlation with root dry mass. The aim of the present study was to improve robustness and efficiency of methods based on capacitance and other electrical variables, such as serial/parallel resistance, conductance, impedance or reactance. Using different electrode configurations and stem contact electrodes, we have measured the electrical impedance spectra of wheat plants grown in pots filled with three types of soil.

**Results:**

For each configuration, parallel capacitance and other linearly independent electrical variables were computed and their quality as root dry mass estimator was evaluated by a ‘sensitivity score’ that we derived from Pearson’s correlation coefficient *r* and linear regression parameters. The highest sensitivity score was obtained by parallel capacitance at an alternating current frequency of 116 Hz in three-terminal configuration. Using a clamp, instead of a needle, as a stem electrode did not significantly affect the capacitance measurements. Finally, in handheld LCR meter equivalent conditions, capacitance had the highest sensitivity score and determination coefficient (*r*^2^ = 0.52) at 10 kHz frequency.

**Conclusion:**

Our benchmarking of linear correlations between different electrical variables and root dry mass enables to determine more coherent practices for ensuring a sensitive and robust root dry mass estimation, including in handheld LCR meter conditions. This would enhance the value of electrical capacitance as a tool for screening crops in relation with root systems in breeding programs.

**Electronic supplementary material:**

The online version of this article (doi:10.1186/s13007-016-0133-7) contains supplementary material, which is available to authorized users.

## Background

Higher cereal yield are needed to feed a growing population in the near future [1]. However, partly as a consequence of climate change, yield of cereals tends to level off in different parts of the world as crops are facing more often sub-optimal conditions (water, nutrients, temperature) for growth [2]. The root system is a central actor in alleviating stress when inputs are suboptimal or limiting [3] and, in such conditions, water/nutrient capture is directly linked to root distribution and activity in soil in relation with the temporal pattern of resource availability. For example, depending on rainfall pattern, root proliferation in shallow soil layer may exhaust soil water too quickly before anthesis, detrimentally to yield, while deep rooting would be advantageous in capturing deep stored water at post-anthesis, and beneficial to yield [4–7]. Both modelling and field experiments showed that such a deep rooting could indeed increase or maintain grain yield for rainfed wheat [8, 9]. Nevertheless, regardless of the root distribution pattern, increase/decrease in root density and biomass would be a factor influencing yield, depending on the environmental conditions [6, 7]. Besides, the early vigour and growth of root systems also plays a major role in drought tolerance, as shown for barley [10]. Whereas high-throughput phenotyping is developing extensively [11, 12], especially on aerial parts, root system traits’ estimation is still time-consuming, expensive in terms of manpower and highly destructive [13]. In the context of crop selection based on root traits, including greenhouse and field selection stages [14], fast techniques for root systems characterization are relevant and needed [15]. Fast imaging techniques [16] and methods based on the root electrical properties could shorten greenhouse selection stages, but at the cost of limited pot size and varying measurement reliability, respectively.

A linear correlation between root mass and electrical capacitance has been empirically found [17] and, later, an equivalent electrical model has been proposed [18]. The capacitance measurement has been tested for different plants under greenhouse condition, for different potting conditions: soil, potting mix substrates, hydroponics, pots of different sizes [19–24]. It has been argued that the correlation between capacitance and root mass comes from allometric relationships in hydroponics [25, 26]. However, such electrical measurements were also successfully experimented these last years in the field for root length density estimation [27] and root trait selection in wheat [28]. In addition, 3D capacitance tomography has been used as a root distribution probe in laboratory [29].

Only few studies [17, 22] were aimed at improving the efficiency, practicability and sensitivity of root biomass estimation with electrical methods. The response of electrical methods applied to soil can be affected by environmental factors (soil water content, temperature, salinity) [30]. However, for soil–plant applications of electrical methods, the optimal experimental setup of measurement remains to be determined: current frequency and voltage, the number of terminals, the electrode type and the electrical variable used as a root mass estimator.

In this study, we propose to (1) evaluate the correlation between root dry mass (RDM, in g) and parallel capacitance over a frequency range of 0.5–20,000 Hz, (2) measure the impact of electrode-stem contact on capacitance values, (3) measure the impact of terminal number on the coefficient of determination between root dry mass and capacitance, (4) compare different widely used electrical variables and (5) evaluate the accuracy of handheld equivalent LCR meter measurements.

## Theory

### Electrical parameters and equivalent RC circuits

In alternating current circuits, electrical impedance is an extension of the concept of resistance in Ohm’s Law. Impedance is defined by two parameters, which are measured with an LCR meter: (1) the magnitude Z (equal to the ratio U/I, where U and I are the sinusoidal voltage and current amplitudes respectively), and (2) the phase angle θ (which expresses the phase shift between sinusoidal tension and current or equivalently the time difference between the maxima of sinusoidal current and tension). The variation of these two parameters with respect to frequency is the impedance spectrum. Impedance $\rm{Z}^{*}$ is a complex number that describes the effect of the circuit on both the magnitude and phase of the electrical signal. In complex notation, impedance can be decomposed in its real (in phase) and imaginary (out of phase) part as follow:

1 $${Z^{*} = Z \times e^{j\theta } = Z \times \cos (\theta ) + j \times Z \times \sin (\theta )}$$

where ${j = \sqrt { - 1} }$. However, other electrical descriptions can also be used, assuming that the equivalent circuit of the investigated system is serial or parallel. In the case of serial circuits, classical simple variables would be either the resistance R (Eqs. 2, 3) and reactance X (Eqs. 2, 4) which are respectively the real and imaginary parts of impedance (both in Ω), or expressed as elements of a serial RC circuit: the serial resistance R_s_ (in Ω, Eq. 6) and serial capacitance C_s_ (in farads, Eq. 7) of. All these electrical variables can be expressed as functions of Z and θ, as follow:

2 $${Z^{*} = R + j \times X}$$

3 $${R = Z \times \cos (\theta )}$$

4 $${X = Z \times \sin (\theta )}$$

5 $${Z = R_{s} + \frac{1}{{j \times \omega \times C_{s} }}}$$

6 $${R_{s} = Z \times \cos (\theta )}$$

7 $${C_{s} = \frac{ - 1}{\omega \times Z \times \sin (\theta )}}$$

${\omega = 2\pi f}$ is the angular frequency, with *f* frequency of injected current.

In the case of parallel circuits, classical variables would be the conductance G (Eq. 10) and susceptance B (Eq. 11), both in Siemens, which are the real and imaginary part of admittance ${\rm{Y}}^{*}$ (inverse of impedance, Eq. 8, in Siemens), respectively. The parallel resistance R_p_ (in Ω) and parallel capacitance C_p_ (in farads) of the parallel RC equivalent circuit are given by Eqs. 13 and 14.

8 $${Y^{*} = \frac{1}{{Z^{*} }} = G + j \times B}$$

9 $${Y = \frac{1}{{Ze^{j\theta } }}}$$

10 $${G = \frac{\cos (\theta )}{Z}}$$

11 $${B = \frac{\sin (\theta )}{Z}}$$

12 $${\frac{1}{{Z^{*} }} = \frac{1}{{R_{p} }} + j \times \omega \times C_{p} }$$

13 $${R_{p} = \frac{Z}{\cos (\theta )}}$$

14 $${C_{p} = \frac{ - \sin (\theta )}{\omega \times Z}}$$

Each of these interlinked variables could be tested for a link with plant roots. For simplifying the choice and number of electrical variables to be studied in relation with plant roots, we consider only those that are neither equal nor proportional to each other. Thus, we can discard R which is equal to R_s_, and B which is proportional to C_p_.

### Effect of injected current frequency

A classic RC parallel circuit (i.e. a circuit with a constant capacitor C and a constant resistor R in parallel) displays a constant value of C_p_ and R_p_ over the whole spectrum (i.e. with any test signal frequency). Likewise, a RC serial circuit (i.e. a circuit with a constant capacitor C and a constant resistor R in series) displays a constant value of C_s_ and R_s_ over the whole spectrum. These simple circuits can be described by one or two electrical variables that are constant with respect to frequency.

However, in complex systems like biological entities, the electrical variable measured can show frequency dependence (e.g., measured C_p_ varies with frequency). Such variations point to a more complex equivalent electrical circuit than a simple lumped RC circuit.

Furthermore, a non-linear behaviour of an electrical variable with frequency implies that comparison of two investigated systems is also frequency dependant. For example, if one uses an electrical parameter as an explanatory variable of the mass a plant root system, conclusions of comparative studies of two plants will depend on the frequency. A ratio of these two electrical parameters computed at a given frequency will differ from a ratio of these parameters computed at another frequency. As a consequence, measurements performed at different frequencies are not equivalent.

### Electrode configuration in impedance measurements

Impedance measurements can be done with different electrode configurations, which are more or less sensitive to bias. Four-terminal (4T) sensing is a technique that eliminates the electrodes’ contact impedance from measurement. This is achieved by separating pairs of current injection electrodes (C1 and C2) and voltage-measuring electrodes (P1 and P2). However, most measurements on plants are performed in a two-terminal (2T) configuration, where current and voltage-measuring electrodes are merged, leading to C1–P1 and C2–P2 electrode patterns. This configuration is sensitive to contact impedance. Finally, an intermediate configuration with three terminals (3T) is made possible by merging a current electrode and a voltage-measuring electrode (e.g., merging C1 and P1), analogous to ground resistance measurement.

## Methods

### Soil and plant material

We used three soil types of contrasting textures: a silt loam (20.9 % sand, 53.3 % silt and 25.8 % clay), a loam (37.7 % sand, 48.7 % silt and 13.6 % clay) and a sandy loam (60.4 % sand, 26.6 % silt and 12.9 % clay). Plastic pots (12.5 cm × 12.5 cm × 22 cm) were filled with 2.5 dm^3^ of these air-dry soils, over a coarse sand and gravel layer for drainage. The field capacity of pots for the different potting substrates was estimated before sowing. Pots were watered with 500 cm^3^ of tap water. Three seeds of durum wheat (cv Isildur) were planted in each pot and the pots were transferred into a growth chamber maintained at 25 °C. Twenty-four hours after sowing, 100 cm^3^ of tap water were added. After emergence, plants were brought to a greenhouse. The pots were thinned to one seedling per pot about 1 week after emergence. In the course of plant growth, pots were weighted regularly (each 2–3 days) and water added to reach the estimated field capacity. Electrical measurements in pots containing silt loam soil were performed 15, 21, 30, 37, and 45 days after sowing, with 4, 2, 2, 2, 2 and 4 replicates, respectively. Measurements for pots containing loam and sandy loam substrates were performed 15, 30, 38 and 45 days after sowing, with 2 replicates for each sampling date. After electrical measurement completion, root systems were collected by carefully washing off the soil and collecting roots on 0.5 mm and 2 mm sieves. The roots were oven-dried at 65 °C for 24 h and their dry masses were precisely recorded on an electronic scale.

### Measurement of the electrical impedance spectrum

Electrical impedance was measured with a SIP FUCHS III LCR-meter (Radic Research, Germany) at 26 logarithmically distributed, pre-programmed current frequencies, ranging from 0.5 to 20,000 Hz, with 1 V terminal voltage. This device enables measurements with 2 terminals (2T), 3 terminals (3T) and 4 terminals (4T) configuration. The electrical variables delivered by the SIP FUCHS III are the magnitude of impedance (*Z*) and the phase angle (*θ*).

### Tests of different terminal configurations

**Fig. 1:**
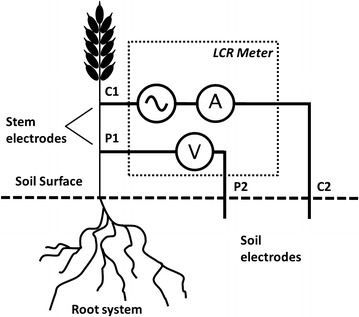
Four-terminal (4T) sensing of plant–soil system. *P1* and *P2* are voltage-measuring electrodes. The LCR meter is simplified by an alternating current source, an ammeter A measuring current flowing between *C1* and *C2* electrodes and a voltmeter V measuring voltage between *P1* and *P2* electrodes. Phase difference between measured current and measured voltage is also performed by the LCR meter. Three-terminal (3T) sensing used in this study is obtained by merging *C1* and *P1*, and conventional two-terminal (2T) sensing is obtained by additionally merging *C2* and *P2*

This first experiment involved 30 wheat plants. Those plants were successively measured in 2T, 3T and 4T configurations. The electric circuit (Fig. 1) includes an alternating current source (electrodes C1 and C2 inserted into the plant and soil, respectively) and voltage was measured between electrodes P1 and P2. In each configuration, electrode C1, an alligator clamp with 15-mm clamp width, was placed on the stem and maintained precisely 5 cm above the soil surface, and 10 cm long bronze rods (diameter 1 mm) were used as soil terminal electrodes, inserted 3 cm deep into the potting soil. For 2T experiments, electrodes C1–P1 (plant) as well as C2–P2 (soil) were merged. Soil terminal electrode C2–P2 was positioned 8 cm away from the stem base. For 3T experiments, plant electrodes C1–P1 were merged, and soil terminal electrodes P2 and C2 were positioned 4 cm and 8 cm away from the stem base, respectively. Finally, for 4T experiments, the plant terminal electrode P1 was placed few millimetres above the soil surface, and the soil terminal electrodes P2 and C2 were positioned 4 cm and 8 cm away from the stem base, respectively.

### Comparison between different electrode types

In a second experiment, 30 wheat plants were subjected to plant-electrode contact comparison. When stems were high enough, a stainless steel alligator clamp with 15 mm clamp width and a 0.5 mm diameter stainless steel needle were successively used as plant contact electrodes for measurements. From those data, the relative difference Δ*C*_*p*_ between *C*_*c*_ and *C*_*n*_, the parallel capacitances obtained with alligator clamp and needle, respectively, was calculated by |*C*_*c*_ − *C*_*n*_|/*C*_*c*_. To determine statistical significance of Δ*C*_*p*_, the distributions of capacitances (*C*_*n*_ and *C*_*c*_) were compared using the Wilcoxon signed-rank test, with an α error of 0.01.

### Comparison between different potting soils

The third experiment involved 30 plants grown in pots filled with different soils (14 plants grown in silt loam, 8 in loam, 8 in sandy loam). The impacts of the three soil types on the coefficient of determination between root mass and electrical capacity have been compared.

### Electrical variables considered

For each measurement, magnitude (Z, in Ω) and phase angle (θ, in °) of electrical impedance were obtained from SIP FUCHS III. From these basic complex parameters, different widely used electrical variable were computed [from Eqs. (4), (6), (7), (10), (13) and (14)]: Parallel capacitance (C_p_, in F), serial capacitance (C_s_, in F), parallel resistance (R_p_, in Ω), serial resistance (R_s_, in Ω), conductance (G, in S) and reactance (X, in Ω).

### Quality ranking of root mass predictors

A linear correlation (*y* = *a RDM* + *b*) between root dry mass (RDM) and each electrical variable (*y*) has been calculated for each of the 26 measurement frequencies. Computations of linear model parameters (slope *a* and *y*-intercept *b*) and coefficient of determination *r*^2^ were performed using Matlab (‘nlinfit’ routine). The maximum determination coefficient between an electrical variable and root dry mass is denoted as *r*_*max*_^2^ (dimensionless) and the corresponding frequency, *f*_*max*_ (Hz), is called ‘maximum determination frequency’. Ideal sensors are designed to deliver a response proportional to the measurand [31]. This linear behaviour between input and output ensures: (1) a constant sensitivity free of saturation effects, and (2) a reduced static error, i.e. linearity prevents additional error issued from a conversion of non-linear input signal to linear output. With a constant sensitivity in the measurement range, a linear response will best allow the comparison between two measurements.

However, the determination coefficient *r*^2^ is not a sufficient criterion for rating the efficiency of a root mass predictor. Another key criterion is the sensitivity to a variation of the estimated root mass. In other words, the ratio between two different root masses should ideally equal the ratio of two measurements of an electrical variable. For linear correlation, it is equivalent to a negligible value of the ratio between interception and slope for a characteristic mass of root.

For two given root masses *m*_*1*_ and *m*_*2*_, two measurements of an electrical variable are obtained (*y*_1_ and *y*_2_), thus:

15 $${\frac{{y_{1} }}{{y_{2} }}=\frac{{a \times m_{1} + b}}{{a \times m_{2} + b}}}$$

For a given characteristic root mass, i.e. the order of magnitude of the average root mass measured, we can state:

$${m_{ 1} = \alpha \times m_{ 0} }\;{\text{and}}\;{m_{2} = \beta \times m_{0} }\;{\text{with}}\;{\alpha \approx 1}\;{\text{and}}\;{\beta \approx 1}.$$

Thus Eq. (15) becomes:

16 $${\frac{{y_{1} }}{{y_{2} }}=\frac{{\alpha + \frac{b}{{a \times m_{0} }}}}{{\beta + \frac{b}{{a \times m_{0} }}}}}$$

17 $${\text{If}}\;\left| {\frac{b}{{a \times m_{ 0} }}} \right|{ < < 1}$$

Equation (16) becomes: ${\frac{{y_{1} }}{{y_{2} }}=\frac{\alpha }{\beta }=\frac{{m_{1} }}{{m_{2} }}}$, which is the true ratio between the root masses. Thus, as shown in Eq. (17) when this ratio become close to 1 or is higher, the sensitivity of electrical variable is poor, and it makes the electrical variable unreliable for comparison of root masses.

In order to rank the electrical variables tested as predictors of root dry mass, we introduced a ‘sensitivity score’ *s*, calculated as follows:

18 $${s = max\left( {r_{\rm max }^{2} \times \left( {1 - \left| {\frac{b}{{a \times m_{0} }}} \right|} \right);0} \right)}$$

where *m*_0_ (in g) is a characteristic mass of dry roots, in our case *m*_0_ = 1 g. The sensitivity score represents the accuracy of the comparison between measurements obtained from 2 plants, with the same order of magnitude of root dry mass (*m*_0_). A maximum score (*s* = 1) means that the ratio between two measurements is equal to the ratio between two plants root mass. A minimum score (*s* = 0) means that only very large variations of root mass would be reliably estimated.

### Literature data

Data that we could retrieve from prior experiments on the quantification of plant root biomass using electrical capacitance were compiled [18, 19, 22–25, 32]. Most of these experiments used C_p_ measured in a 2T configuration at 1 kHz for root biomass estimation. The growth media, the measured characteristic biomass of wet or dry roots, the parameters linear regression found between root biomass and C_p_ and the coefficient of determination were extracted in order to computed their respective *y*-intercept:slope ratio (Eq. 17) and their ‘sensitivity score’ *s* (Eq. 18). The measured characteristic biomass of roots was roughly the median of root biomass measured in each experiment.

## Results

### Root mass

The harvested plants presented a root mass ranging from 0.02 to 0.72 g, with a mean value of 0.2 g and a standard deviation of 0.19 g. This reflects the fact that plants were harvested at different times and that they were relatively young (Additional file 1).

### Frequency dependence of the parallel capacitance: root dry mass correlation

**Fig. 2:**
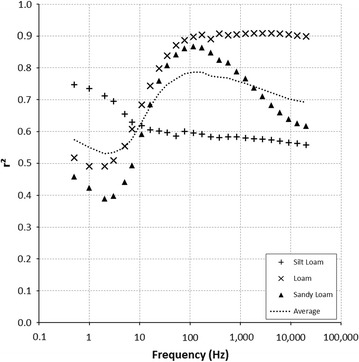
Coefficient of determination (*r* ^2^) between root dry mass and parallel capacitance, as a function of frequency. The semi-log plot was obtained from measurements for a 3T configuration, a frequency range of 0.5–20,000 Hz (log scale). Plants were grown in silt loam (*plus symbol*), loam (*times symbol*) and sandy loam (*filled triangle*) soils. The *black dots* represent the average of the three soil types. The maximum determination frequency *f*_*max*_ for average is 116 Hz

Determination coefficients from linear correlations between root dry mass and parallel capacitance are plotted in Fig. 2 as a function of frequency. The correlation between C_p_ and RDM is frequency-dependant. The same pattern occurs in 2T and 4T configurations (Additional file 2: Figure S1, Additional file 3: Figure S2). On average for the 3 soil types, the maximum determination value *r*_*max*_^2^ (*p* < 0.01) between root dry mass (RDM) and C_p_ equals 0.787, and occurs at a maximum determination frequency *f*_*max*_ of 116 Hz. In this configuration, the RDM (in g) relation with C_p_ (in nF) is:

$${C_{p} = 4.2 \times RDM + 0.37}$$

The magnitude of *r*^2^ at *f*_*max*_ depends on the soil. Thus, at *f*_*max*_ = 116 Hz, the determination coefficient from the linear correlation between biomass and C_p_ in a loam soil reaches high value (*r*^2^ = 0.898), while a silty loam soil has a lower maximum *r*^2^ value of 0.595. In addition, each soil type displays different *f*_*max*_ value, in all terminal configurations.

### Effect of plant electrode type: clamp versus needle electrode on average for the three soil types

**Fig. 3:**
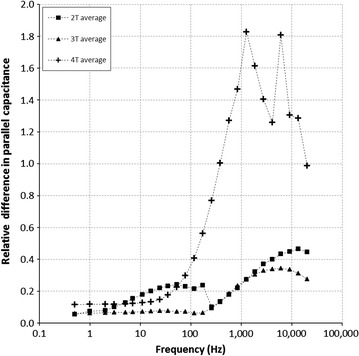
Impact of the stem contact electrode on the measured electrical capacitance of the plant–soil system. Plot of the mean relative difference between the parallel capacitance measured with a clamp and that measured with a needle, as a function of frequency, in 2T (*filled square*), 3T (*filled triangle*) and 4T (*plus symbol*) configurations. Differences are <6 % for frequencies <200 Hz in 3T configuration

The mean relative differences in parallel capacitance Δ*C*_*p*_, between needle measurements and clamp measurements, are shown in Fig. 3. As a general trend, the relative difference Δ*C*_*p*_ tends to increase with the test signal frequency. The relative difference is <20 % over the range of 0.5–100 Hz and is ~6 % for low frequency 2T measurements. However, relative differences in 4T measurements exceed 20 % for frequencies over 100 Hz and peaks to 180 % at 1250 Hz. Nevertheless, 2T and 3T configurations exhibit a relative difference maximum of 50 %, down to 6 %, especially for 3T measurements in the frequency range of 0.5–172 Hz. Additionally, in 2T configuration, the relative difference was not statistically significant (*p* value >0.01) in the frequency range of 0.5–13,458 Hz. The same occurred for 3T and 4T configurations, but in the narrower frequency range of 0.5–381 Hz.

### Performance of electrical variables for root dry mass prediction

Table 1 shows, for all the electric variables studied the sensitivity scores, maximum of coefficient of determination from the linear correlation with root dry mass and maximum determination frequency *f*_*max*_ averaged over the three soil types investigated. An exhaustive list of the regression parameters used for the *s *score calculations are given in Additional file 1 and illustrations of a good, moderate and low score are provided in Additional files 4, 5 and 6 respectively. Parallel capacitance C_p_ reaches a maximum *r*^2^ value with RDM in 3T configuration (*r*_*max*_^2^ = 0.787), while it exhibits a slightly lower maximum *r*^2^ value (*r*_*max*_^2^ = 0.771) in 2T configuration. In 3T configuration, all electrical variables display a *r*_*max*_^2^ close to 0.7, except for the phase angle (*r*_*max*_^2^ = 0.331). However, *f*_*max*_ differs for all variables, ranging from 3 to 20,000 Hz in 2T, 116 to 20,000 Hz in 3T and 0.5 to 20,000 Hz in 4T configuration. Regarding sensitivity scores, the parallel capacitance C_p_ displays the best score in 3T configuration (*s* = 0.717), followed by serial capacitance C_s_ (*s* = 0.688) and conductance G (*s* = 0.679). For all variables except phase angle θ, sensitivity scores were higher in 3T than in 2T and 4T configurations. In general, our sensitivity score changes the ranking of electrical variables by their determination coefficient with root dry mass. In all terminal configurations, C_p_, C_s_ and G exhibit the best sensitivity scores, especially in 4T measurement where R_p_, R_s_, Z and X scores are null, meaning that these variables are not reliable for root mass estimation.

**Table 1:** Signal frequencies leading to the highest scores for electrical variable depending on the chosen terminal configuration

Electrical variable	Terminal configuration	Sensitivity score	*r*_*max*_^2^	*f*_*max*_ (Hz)
C_p_	2T	0.71	0.771***	78
	3T	0.72	0.787***	116
	4T	0.45	0.560	566
C_s_	2T	0.48	0.520**	3
	3T	0.69	0.754***	6094
	4T	0.28	0.353	20,000
R_p_	2T	0.44	0.657***	78
	3T	0.53	0.797***	841
	4T	0.00	0.377	9056
R_s_	2T	0.45	0.642**	9056
	3T	0.54	0.791***	4101
	4T	0.00	0.348	20,000
G	2T	0.52	0.585**	78
	3T	0.68	0.751***	4101
	4T	0.31	0.398	20,000
Z	2T	0.43	0.645***	78
	3T	0.53	0.795***	1857
	4T	0.00	0.357	1,3458
X	2T	0.46	0.653**	20,000
	3T	0.55	0.794***	6094
	4T	0.00	0.330	20,000
θ	2T	0.05	0.331	20,000
	3T	0.10	0.752***	20,000
	4T	0.26	0.374	0.5

The sensitivity scores, the maximum of the coefficient of determination with root dry mass (*r*_*max*_^2^) and the maximum determination frequency (*f*_*max*_, in Hz), for parallel capacitance (C_p_), serial capacitance (C_s_), parallel resistance (R_p_), serial resistance (R_s_), conductance magnitude (G), impedance magnitude (Z), reactance (X) and phase angle (θ), in 2T, 3T and 4T configurations averaged for the three soil types studied

*** Linear regression is significant at the 0.01 level

** Linear regression is significant at the 0.05 level

### Performance of terminal configurations for root dry mass estimation

The number of terminals is generally related to the coefficient of determination from linear correlations between electrical variables and RDM (Table 1). The 3T measurements exhibit the best *r*^2^ values, while 2T measurements have slightly lower, yet close, *r*^2^ values. Four-terminal configuration shows the worst determination of RDM, with *r*^2^ < 0.5. Parallel capacitance (C_p_), C_s_ and G maximum determination frequencies increase with the number of terminals. The 2T (see Additional file 2) and 3T measurements exhibit similar *y*-intercept/slope ratios of the linear regression, but lower than that of 4T measurements (see Additional file 3). This means that this latter configuration is less sensitive to the root biomass variations. Most of experiments relating root biomass and electrical capacitance in the literature were performed with handheld LCR meters in 2T configuration. Most of the time, the test signal frequency used is 1000 Hz, but 100 and 10,000 Hz frequencies are also available on LCR meters. Table 2 shows the results of our experiments close to these measurement conditions: frequencies considered are 116, 1250 and 13,458 Hz with 2T configuration.

**Table 2:** Sensitivity scores for each electrical variable in conditions similar to the widely used LCR meters

Electrical variable	Frequency (Hz)	Sensitivity score	*r* ^2^
C_p_	116	0.49	0.542
	1250	0.46	0.524
	13,458	0.50	0.605
C_s_	116	0.10	0.119
	1250	0.29	0.331
	13,458	0.38	0.436
R_p_	116	0.40	0.584**
	1250	0.45	0.635**
	13,458	0.38	0.599**
R_s_	116	0.39	0.563**
	1250	0.44	0.615**
	13,458	0.44	0.639**
G	116	0.28	0.317
	1250	0.35	0.394
	13,458	0.45	0.502
Z	116	0.39	0.574**
	1250	0.44	0.628**
	13,458	0.41	0.622**
X	116	0.18	0.281
	1250	0.42	0.595**
	13,458	0.46	0.650**
Θ	116	0.00	0.197
	1250	0.00	0.214
	13,458	0.03	0.267

The sensitivity scores, coefficients of determination with root dry mass (*r* ^2^), for each electrical variable in LCR meter conditions (test signal frequencies of 116, 1250 and 13,458 Hz, in 2T configuration). The highest sensitivity score is obtained by parallel capacitance (C_p_) at 13,458 Hz

*** Linear regression is significant at the 0.01 level

** Linear regression is significant at the 0.05 level

Among electrical variables, parallel capacitance displays the highest sensitivity score (*s* = 0.50) of all variables at 13,458 Hz. Considering only determination coefficients, and thus ignoring the sensitivity, reactance reaches the highest coefficient of determination (*r*^2^ = 0.649), this makes it the best choice. Interestingly, at 1250 Hz frequency, parallel capacitance reaches the highest sensitivity over all other variables. However its sensitivity score at 1250 Hz (*s* = 0.46) is lower than at 13,458 Hz (*s* = 0.50). This would make C_p_ measured at 13,458 Hz, the best choice in terms of sensitivity and precision for root biomass estimation in 2T configuration with handheld meters.

### Effect of the growth media on root electrical relationship: data from literature

**Fig. 4:**
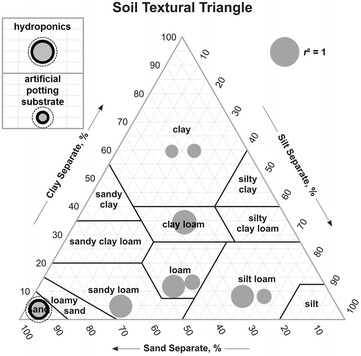
Growth media used in root biomass estimation found in related literature and in our study. The size of the *circles* is proportional to the coefficient of determination *r* ^2^ found in each study. When more than two studies involved the same growth media, the mean (in *black ring*), the minimum (in *grey circle*) and the maximum (*dashed circle*) of coefficient of determination *r* ^2^ were plotted

The sensitivity scores *s* obtained by previous studies are shown in Table 3 and the determination coefficients *r*^2^ obtained in different growth media (including soils, potting substrates, hydroponics) are shown Fig. 4. The highest *s* and *r*^2^ values are obtained in hydroponics (*s* = 0.99–0.64, *r*^2^ = 0.99–0.67), whereas artificial potting substrate (vermiculite, compost, sheep manure) had the lowest *s* values (*s* = 0.30–0.00). The experiments involving natural soils exhibited intermediate *s* values (*s* = 0.51–0.36), and strong *r*^2^ values (*r*^2^ = 0.82–0.50). Few data involve clay soils, while most studies focused on sandy to loamy soils.

**Table 3:** Compilation of linear regression parameters between parallel capacitance and root mass, and corresponding sensitivity scores from literature data

Publication	Species	Characteristic root mass *m*_*0*_ (g)	*b*/(*a* × *m*_*0*_)	*r* ^2^	*s*	Growth media	Comments
Chloupek [17]	*Zea mays*	–	–	0.728	–	Sand	Dried
	*Allium cepa*	–	–	0.545	–	Sand	Dried
	*Helianthus annuus*	–	–	0.897	–	Sand	Dried
	*Avena sativa*	–	–	0.464	–	Clay soil	Dried
	*Helianthus annuus*	–	–	0.432	–	Clay soil	Dried
	*Brassica napus*	–	–	0.081	–	–	Fresh
Chloupek [37]	*Daucus carota*	–	–	0.514	–	Loam (field)	Fresh
	*Helianthus annuus*	–	–	0.566	–	Sand	Fresh
Kendall et al. [32]	*Medicago sativa*	0.2	0.03	0.50	0.48	Silt loam (field)	Dried
	*Trifolium Pratense*	–	–	0.67	–	Hydroponics	Dried
Dalton [18]	*Solanum lycopersicum* Mill.	2	0.17	0.77	0.57	Hydroponics	Dried
van Beem et al. [24]	*Zea mays* L.	100	0.17	0.53	0.44	Loam (field)	Fresh
		5	1.33	0.73	0.00	Vermiculite	Fresh
Ozier-Lafontaine and Bajazet [23]	*Solanum lycopersicum* Mill.	1	0.55	0.82	0.36	Clay loam	Dried
	*Solanum lycopersicum* Mill.	1	0	0.99	0.99	Hydroponics	Dried
Aulen and Sipley [19]	Herbaceous species	0.1	0	0.30	0.3	Compost	Dried
Dietrich et al. [25]	*Triticum aestivum* L.	1	0.32	0.75	0.51	Sand	Dried
Ellis et al. [36]	*Vicia faba* L.	10	0.48	0.31	0.16	Sheep manure	Fresh
Present study	*Triticum turgidum* L. ssp. *durum*	1	0.09	0.787	0.72	Silt loam, loam, sandy loam	Dried

## Discussion

This work shows that the accuracy of electrical variables used for predicting biomass is frequency-dependent. The best estimates were obtained within a specific frequency range, using parallel capacitance as a proxy for biomass estimator. Our experiments, like others from the literature, involve plant and soil electrical probing. In other words, soil electrical response is also measured and can interfere with plant response. Thus regarding the parallel capacitance, the frequency range where the *r*^2^ values are low, may correspond to the frequency range where the soil electrical response is stronger than the plant electrical response. Soil texture induces variations of this frequency range (Fig. 2), supporting that the full electrical response spectrum is a combination of soil and plant responses. Furthermore, the frequency dependence of parallel capacitance implies that plant system cannot be simplified to a simple RC parallel circuit. In such circuit, C_p_ is constant over the whole frequency range. This implies that an electrical equivalent of plant–soil system is composed of several parallel RC circuits, exhibiting several relaxation times. This frequency dependence has also been reported on other plants than wheat, such as tomatoes [23].

Among factors affecting the electrical response of this soil–plant method, root type or soil conditions have been shown to interact with the signal. Indeed, woody and non-woody roots may respond differently [33, 34] due to a difference in their inner root structure. Heterogeneity of the growing medium alters the electrical relation: non-homogeneous substrate (e.g. manure or compost) display the lowest coefficients of determination [19, 22, Fig. 4] and in a lesser extent, results may vary from hydroponics to real soils (Fig. 4), and from pot to field experiments [24]. Soil water content appears as the most influential environmental factor [25, 27, 32] and, consequently, as a major constituent in the reliability and accuracy of measurements when it varies. The soil water content effect was minimised in our study, since pots were adjusted at field capacity before measurements. However, this factor will undeniably add noise to the electrical relation if it is variable, particularly in the field [35].

In the range of low frequencies (<200 Hz), for plants like wheat, needle measurements only slightly differ from clamp measurements. This implies that the electrical current path is not mastered by the different contact types of these electrodes. Even if xylem is the best carrier of electrical current, it appears that stem impedance is small over the width of a clamp. Its additive effect on measured impedance is negligible in comparison with whole plant impedance. Furthermore, clamps are less destructive and more practicable than needles and could be preferred for root capacitance measurements. At higher frequencies, needle and clamp measurements can exhibit discrepancies. This is particularly true for the 4T configuration, for which the relative difference between electrode types peaks at 180 %. 4T configuration is generally known for being more reliable. It eliminates contact impedance and enables precise measurements of impedance. Thus, the higher differences between needle and clamp measurements found with 4T, compared with 2T and 3T configurations in our experiment, could seem counter-intuitive. These larger differences found in 4T may have various causes.

Firstly, needle electrodes, implanted trough the stem, are more invasive than clamp electrodes. Perturbation of the flow in the xylem can occur and modify the displacement of the electric charges between electrodes, creating a parasitic effect in the measured medium. This parasitic effect on the charge carrying medium is supported by the lower coefficients of determination with root dry mass found in 4T configuration.

Secondly, it has been shown that measured capacitance is very sensitive to electrode position on the stem [18, 36], in particular for electrode located close to the base of the stem. Our 4T configuration measurements involved electrode contact very close to the base of the stem (few mm). Thus a small deviation on the position of the electrode located at the base of the stem could imply a large difference in the measured capacitance.

In our experiments, 2T and 3T configurations are less sensitive to the stem electrode position. This electrode was located much higher (5 cm), and thus less sensible to a deviation on its position. In consequence, differences found between needle and clamp measurements in 2T and 3T configurations are much lower.

Depending on the type of terminal configuration, the measurements may be biased in different ways. For example, in 2T configuration, measurements could biased by wire and contact impedance, while in 4T configuration, a more representative impedance, eliminating contacts and wire effects, of the device under test would be measured. The 3T configuration is an intermediate configuration, where only contact impedance of the plant electrode is involved. The lower correlations with root dry mass in 4T configuration than in 2T and 3T are probably due to plant electrode issues.

Finally, it appears that classical handheld LCR meter measurements could be revised in terms of frequency and electrical variable used. In the terminal configuration used by this kind of device, i.e. 2T configuration, C_p_ obtained the best sensitivity score at around 10 kHz. However, in these handheld LCR equivalent configurations, sensitivity scores obtained were significantly lower than sensitivity scores obtained with the optimal configuration, i.e. using a 3T configuration at 116 Hz.

As shown Table 3, hydroponics shows the best conditions for root biomass estimation using electrical C_p_. This growth media exhibits an optimal contact with roots as well as homogeneity. In real soils, the root-medium contact is not controlled, the heterogeneity is greatly increased. This results in less precise root biomass estimations, but the electrical-root biomass relationship is still effective. This work enhances root biomass estimations under more representative conditions, by using various real soils instead of hydroponics, providing framework for agronomical relevant root studies.

## Conclusions

In this study, we found that the estimation of wheat root biomass through electrical measurements would be more accurate when performed in 3T configuration with a 116 Hz frequency and using parallel capacitance as the electrical variable. With this measurement setting, low differences (6.7 %) were found when using clamp or needle as plant contact electrode. This result means that reliable measurements can be achieved by using clamps, which are more practicable and less destructive than needles trough stem. We also found that a handheld LCR meter could result in better measurements when used at 10,000 Hz and measuring parallel capacitance, even though reactance obtained better determination coefficients from linear correlation with root dry mass. These methodological optimizations strengthen the robustness of the electrical methodology to assess wheat root biomass and would be useful in pot studies and greenhouse/controlled conditions used in phenotyping. However, application to field trials requires the quantification of the impact of possible interfering factors. Our study was focused on a monocot crop grown in medium sized pots, measured during early growth stages with limited tillering and a moist soil. The electrical relations with root mass and our derived sensitivity score shall be tested with different species (particularly woody or non-woody species) and, more importantly, with contrasted soil water contents.

## Additional files

10.1186/s13007-016-0133-7 Sensitivity score details and linear regression parameters between root dry mass and each electrical variable. The table lists the maximum determination frequency (*f*_*max*_, Hz), sensitivity scores, maximum of coefficient of determination with root dry mass (*r*_*max*_^2^), *y*-intercept of the linear regression (in standard unit of the related electrical variable) and slope (in standard unit of the related electrical variable per g) for parallel capacitance (C_p_), serial capacitance (C_s_), parallel resistance (R_p_), serial resistance (R_s_), conductance magnitude (G), impedance magnitude (Z), reactance (X) and impedance phase angle (θ) in 2T, 3T and 4T configurations, on average for 3 soil types.
10.1186/s13007-016-0133-7 Coefficient of determination (*r* ^2^) between root dry mass and parallel capacitance, in 2T configuration. The semi-log plot of the determination coefficients (*r* ^2^) was obtained over a frequency range of 0.5 to 20,000 Hz (log scale), for plants grown in silt loam (+), loam (×), sandy loam (▲), averaged for the three soil types (black dots).
10.1186/s13007-016-0133-7 Coefficient of determination (*r* ^2^) between root dry mass and parallel capacitance, in 4T configuration. The semi-log plot of the determination coefficients (*r* ^2^) was obtained over a frequency range of 0.5 to 20,000 Hz (log scale), for plants grown in silt loam (+), loam (×), sandy loam (▲), averaged for the three soil types (black dots).
10.1186/s13007-016-0133-7 Illustration of a linear regression with high coefficient of determination and high sensitivity score *s*. Parallel capacitance measured at 116 Hz, rated as the best configuration obtained in this study. Data from plants grown in pots containing loam, measured in 3T configuration.
10.1186/s13007-016-0133-7 Illustration of a linear regression with high coefficient of determination but low sensitivity score *s*. Due to its high *r* ^2^, reactance measured at 20 kHz may turn out to be a good candidate, however its interception is largely greater than zero implying a lowered score. Data from plants grown in pots containing loam, measured in 3T configuration.
10.1186/s13007-016-0133-7 Illustration of a linear regression with low coefficient of determination and low sensitivity score *s*. Low r^2^ coupled with non-negligible intercept. Data from plants grown in pots containing sandy loam, measured in 3T configuration.
