# Supplementary material for: Benchmarking electrical methods for rapid estimation of root biomass
Source: Plant Methods. 2016 Jun 22;12:33. doi: 10.1186/s13007-016-0133-7 (PMC4917982; doi:10.1186/s13007-016-0133-7)
Supplement: Supplementary file 1 — 10.1186/s13007-016-0133-7 Sensitivity score details and linear regression parameters between root dry mass and each electrical variable. The table lists the maximum determination frequency (f max, Hz), sensitivity scores, maximum of coefficient of determination with root dry mass (r max2), y-intercept of the linear regression (in standard unit of the related electrical variable) and slope (in standard unit of the related electrical variable per g) for parallel capacitance (Cp), serial capacitance (Cs), parallel resistance (Rp), serial resistance (Rs), conductance magnitude (G), impedance magnitude (Z), reactance (X) and impedance phase angle (θ) in 2T, 3T and 4T configurations, on average for 3 soil types. [file 13007_2016_133_MOESM1_ESM.docx]

**Table S1.**

| Electrical variable | Terminal configuration | *f_max_* (Hz) | *r²_max_* | Sensitivity score | *y*-Intercept | Slope |
| --- | --- | --- | --- | --- | --- | --- |
| Cp | 2T | 78 | 0.771 | 0.71 | 3.8E-10 | 4.6E-09 |
|  | 3T | 116 | 0.787 | 0.72 | 3.7E-10 | 4.2E-09 |
|  | 4T | 566 | 0.560 | 0.45 | 1.3E-08 | -6.6E-08 |
|  |  |  |  |  |  |  |
| Cs | 2T | 3 | 0.520 | 0.48 | 1.4E-06 | 1.7E-05 |
|  | 3T | 6,094 | 0.754 | 0.69 | 2.0E-10 | 2.3E-09 |
|  | 4T | 20,000 | 0.353 | 0.28 | 7.7E-09 | -3.6E-08 |
|  |  |  |  |  |  |  |
| Rp | 2T | 78 | 0.657 | 0.44 | 6.0E+05 | -1.8E+06 |
|  | 3T | 841 | 0.797 | 0.53 | 3.1E+05 | -9.5E+05 |
|  | 4T | 9,056 | 0.377 | 0.00 | 1.6E+04 | -7.3E+03 |
|  |  |  |  |  |  |  |
| Rs | 2T | 9,056 | 0.642 | 0.45 | 1.9E+05 | -6.4E+05 |
|  | 3T | 4,101 | 0.791 | 0.54 | 2.5E+05 | -8.0E+05 |
|  | 4T | 20,000 | 0.348 | 0.00 | 1.8E+04 | -8.8E+03 |
|  |  |  |  |  |  |  |
| G | 2T | 78 | 0.585 | 0.52 | 1.4E-06 | 1.4E-05 |
|  | 3T | 4,101 | 0.751 | 0.68 | 3.3E-06 | 3.4E-05 |
|  | 4T | 20,000 | 0.398 | 0.31 | 3.4E-04 | -1.5E-03 |
|  |  |  |  |  |  |  |
| Z | 2T | 78 | 0.645 | 0.43 | 6.1E+05 | -1.8E+06 |
|  | 3T | 1,857 | 0.795 | 0.53 | 2.6E+05 | -7.9E+05 |
|  | 4T | 13,458 | 0.357 | 0.00 | 1.6E+04 | -7.3E+03 |
|  |  |  |  |  |  |  |
| X | 2T | 20,000 | 0.653 | 0.46 | -6.3E+04 | 2.2E+05 |
|  | 3T | 6,094 | 0.794 | 0.55 | -1.1E+05 | 3.4E+05 |
|  | 4T | 20,000 | 0.330 | 0.00 | -8.6E+03 | 5.0E+03 |
|  |  |  |  |  |  |  |
| θ | 2T | 20,000 | 0.331 | 0.05 | -35.53 | 41.19 |
|  | 3T | 20,000 | 0.752 | 0.10 | -51.21 | 59.08 |
|  | 4T | 0.5 | 0.374 | 0.26 | -3.23 | 10.20 |
