# Supplementary figures and images for: Benchmarking electrical methods for rapid estimation of root biomass
Source: Plant Methods. 2016 Jun 22;12:33. doi: 10.1186/s13007-016-0133-7 (PMC4917982; doi:10.1186/s13007-016-0133-7)

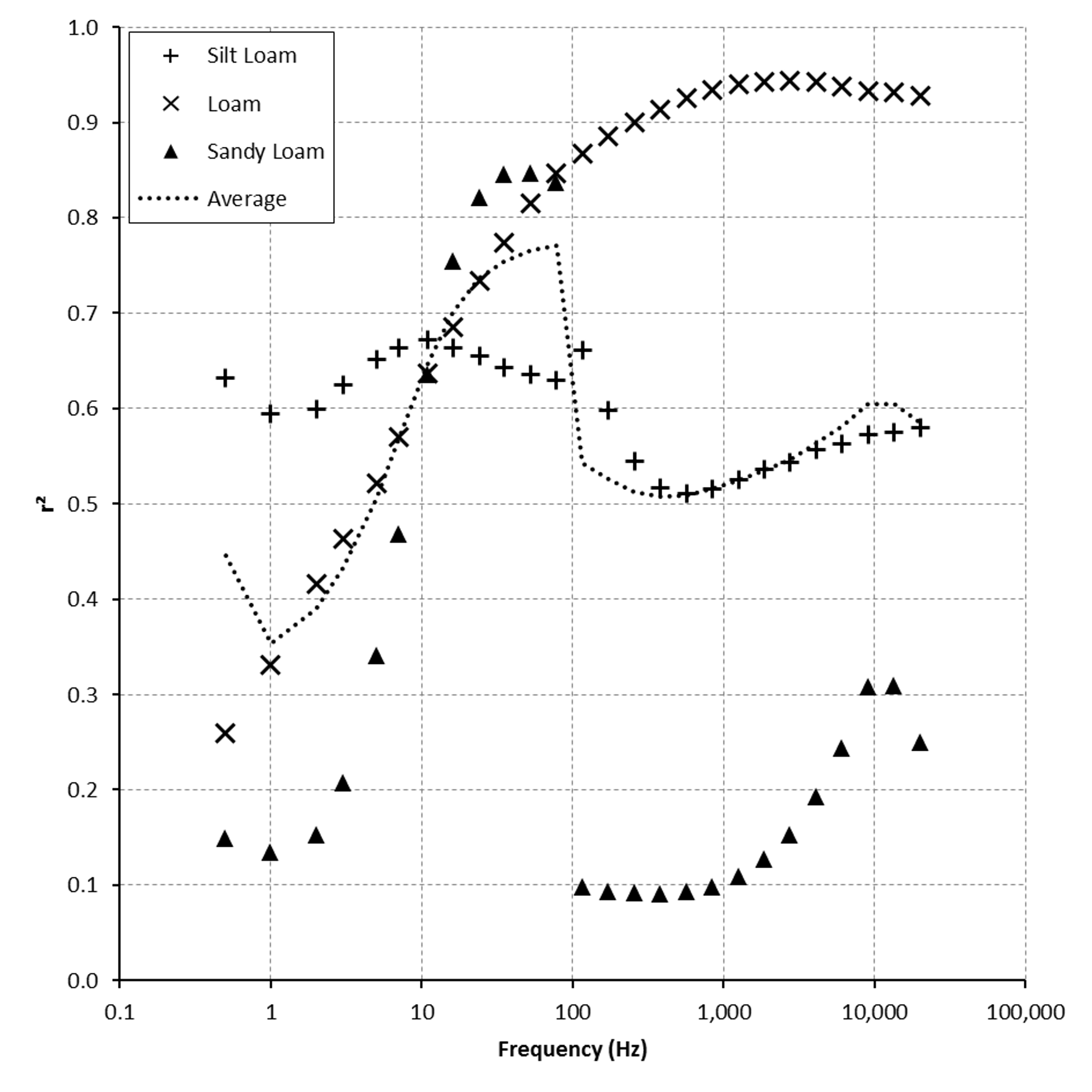

Supplement: Supplementary file 2 — 10.1186/s13007-016-0133-7 Coefficient of determination (r 2) between root dry mass and parallel capacitance, in 2T configuration. The semi-log plot of the determination coefficients (r 2) was obtained over a frequency range of 0.5 to 20,000 Hz (log scale), for plants grown in silt loam (+), loam (×), sandy loam (▲), averaged for the three soil types (black dots). [file 13007_2016_133_MOESM2_ESM.tif]

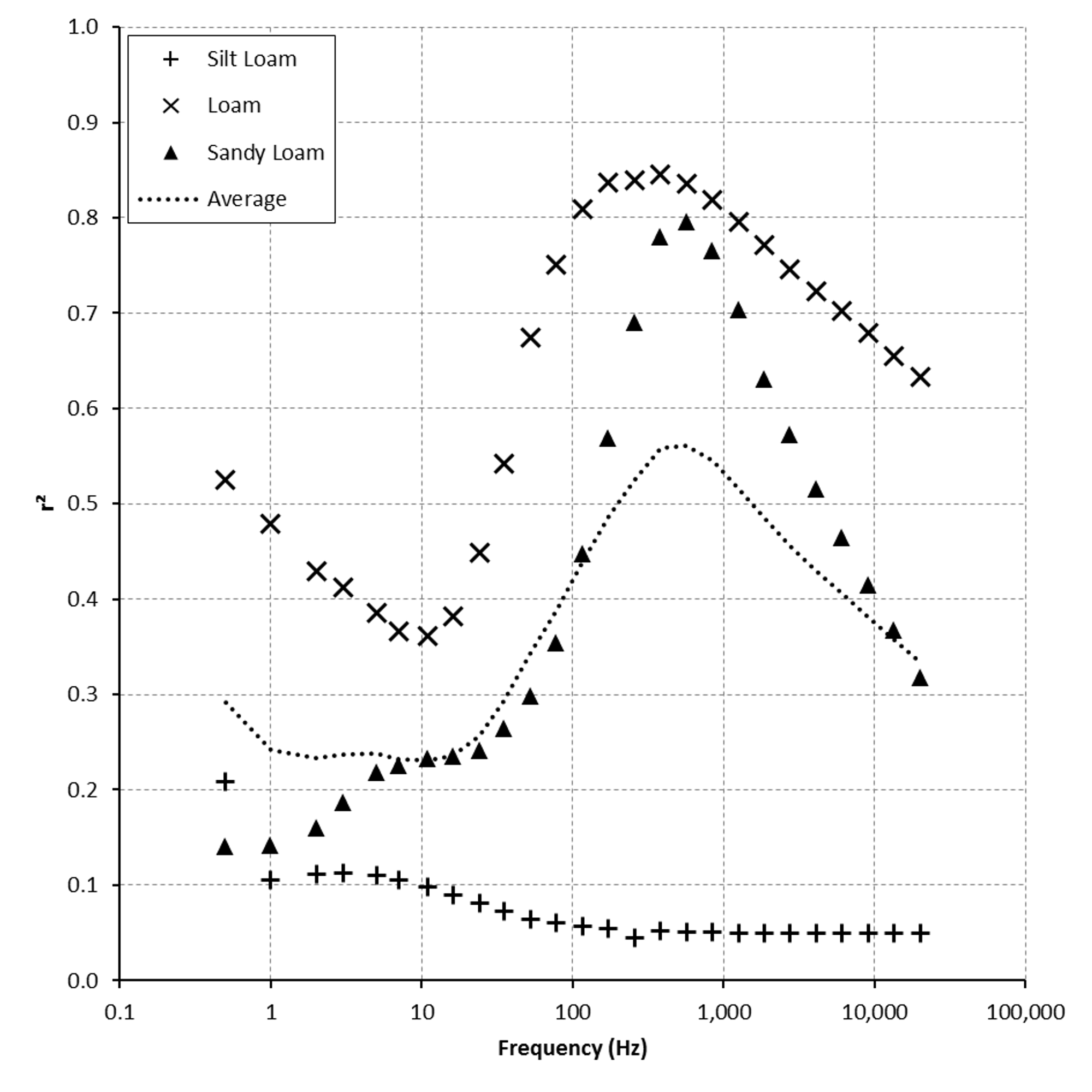

Supplement: Supplementary file 3 — 10.1186/s13007-016-0133-7 Coefficient of determination (r 2) between root dry mass and parallel capacitance, in 4T configuration. The semi-log plot of the determination coefficients (r 2) was obtained over a frequency range of 0.5 to 20,000 Hz (log scale), for plants grown in silt loam (+), loam (×), sandy loam (▲), averaged for the three soil types (black dots). [file 13007_2016_133_MOESM3_ESM.tif]

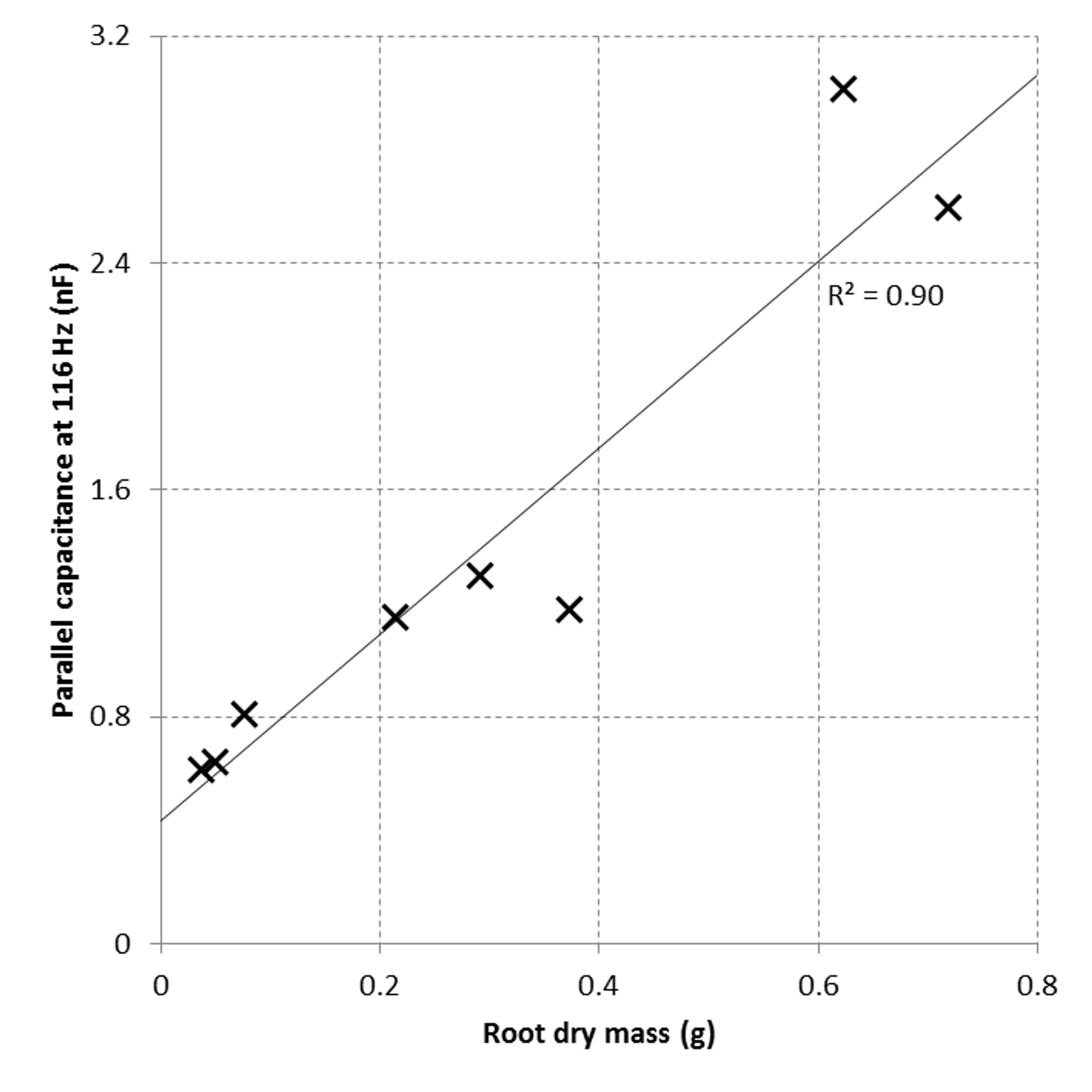

Supplement: Supplementary file 4 — 10.1186/s13007-016-0133-7 Illustration of a linear regression with high coefficient of determination and high sensitivity score s. Parallel capacitance measured at 116 Hz, rated as the best configuration obtained in this study. Data from plants grown in pots containing loam, measured in 3T configuration. [file 13007_2016_133_MOESM4_ESM.tif]

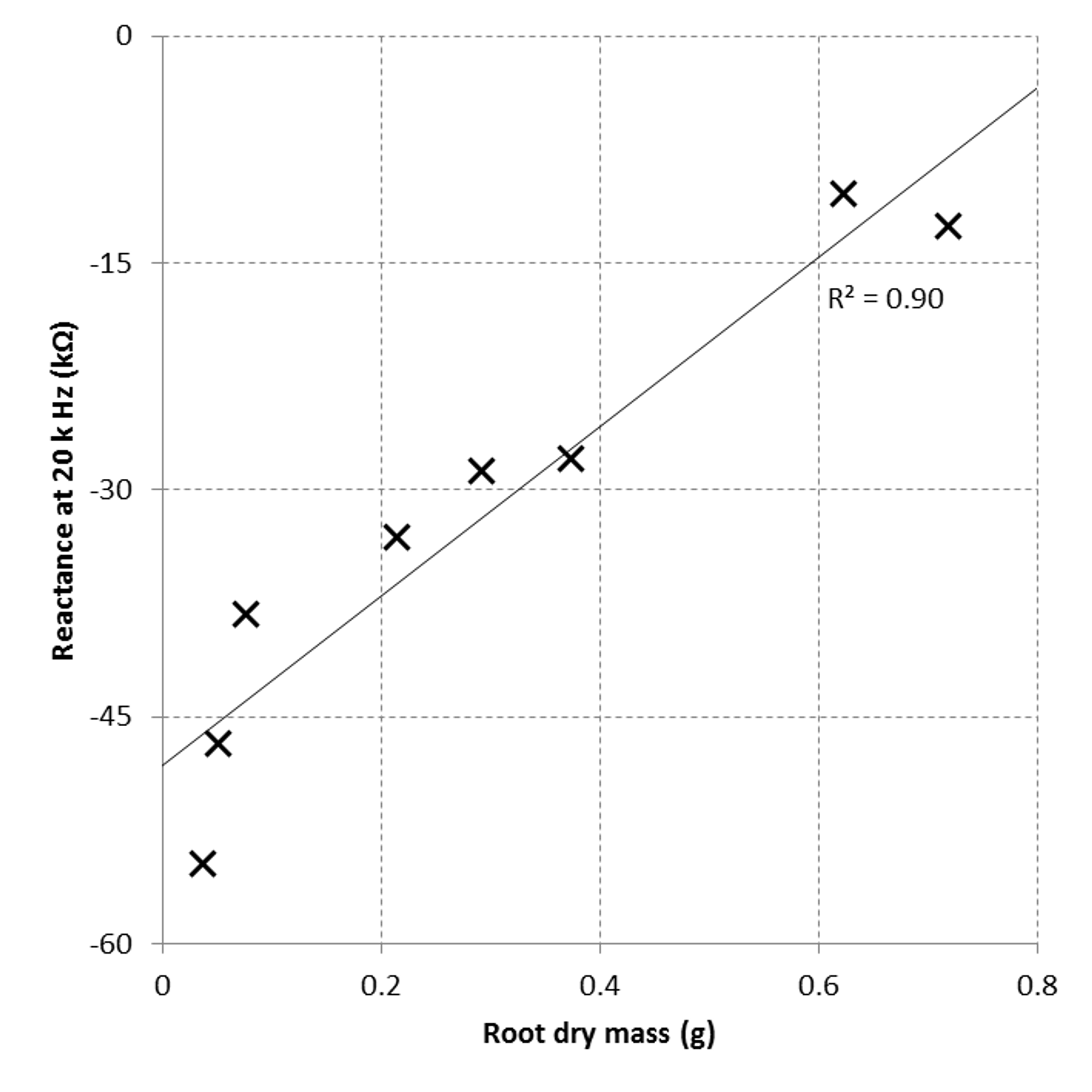

Supplement: Supplementary file 5 — 10.1186/s13007-016-0133-7 Illustration of a linear regression with high coefficient of determination but low sensitivity score s. Due to its high r 2, reactance measured at 20 kHz may turn out to be a good candidate, however its interception is largely greater than zero implying a lowered score. Data from plants grown in pots containing loam, measured in 3T configuration. [file 13007_2016_133_MOESM5_ESM.tif]

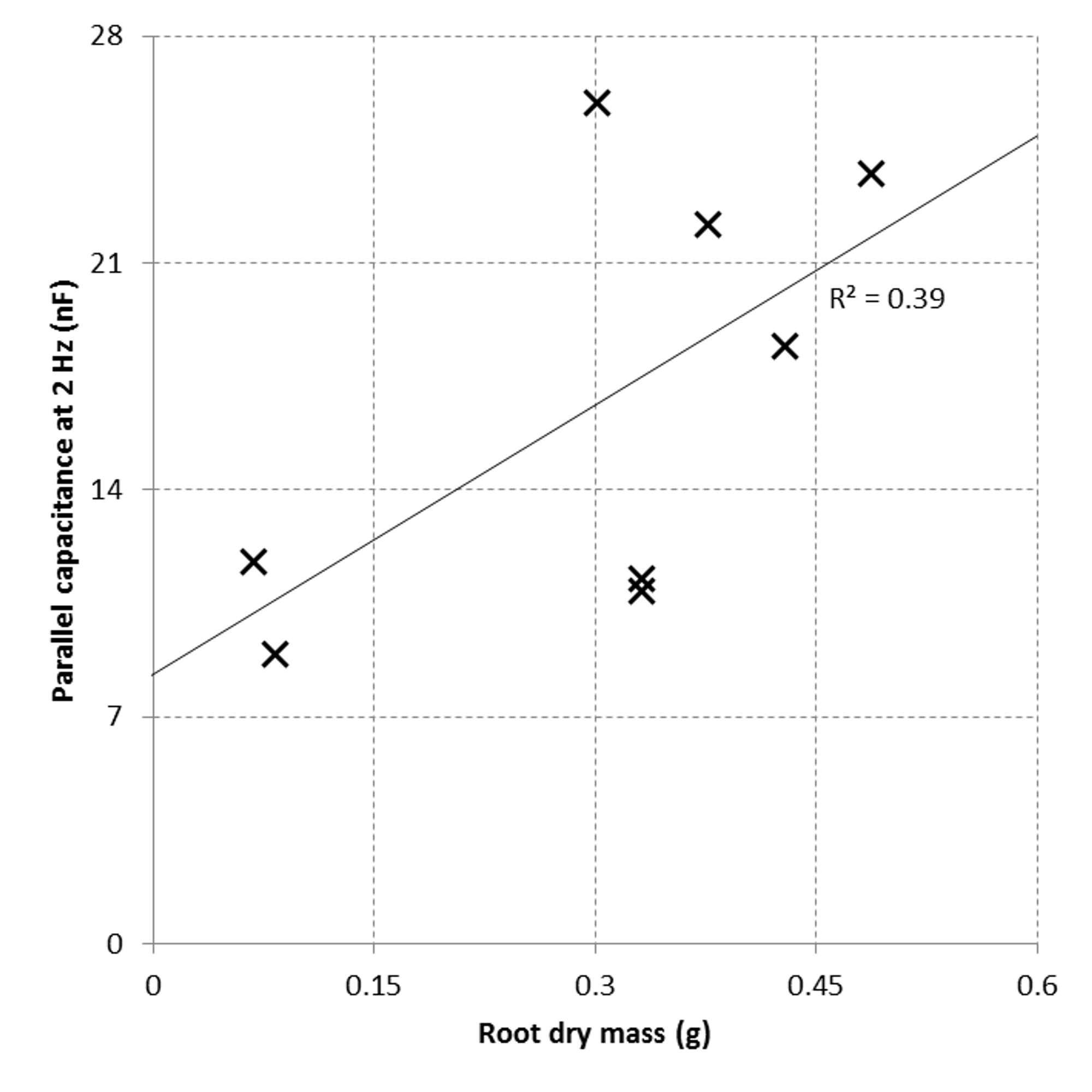

Supplement: Supplementary file 6 — 10.1186/s13007-016-0133-7 Illustration of a linear regression with low coefficient of determination and low sensitivity score s. Low r2 coupled with non-negligible intercept. Data from plants grown in pots containing sandy loam, measured in 3T configuration. [file 13007_2016_133_MOESM6_ESM.tif]
